# Comparison of A and B Starch Granules from Three Wheat Varieties

**DOI:** 10.3390/molecules161210570

**Published:** 2011-12-19

**Authors:** Jie Zeng, Guanglei Li, Haiyan Gao, Zhengang Ru

**Affiliations:** 1 School of Food Science, Henan Institute of Science and Technology, Xinxiang 453003, China; Email: zengjie623@163.com (J.Z.); lgl70_hist@163.com (G.L.); gaohaiyan127@163.com (H.G.); 2 Wheat Research center, Henan Institute of Science and Technology, Xinxiang 453003, China

**Keywords:** wheat starch, A-granule, B-granule, XRD, thermal property, pasting property, FTIR, SEM

## Abstract

Three starches from the wheat varieties AK58, ZM18 and YZ4110 were separated into large (A) and small (B) granules, which were characterized structurally and evaluated for their functional properties. SEM results showed that the size of A-granules from ZM18 and YZ4110 were about the same, but the sizes of A-granules and B-granules from AK58 were larger than those of ZM18 and YZ4110. FTIR spectra showed that all the samples exhibited a similar pattern, with seven main modes with maximum absorbance peaks near 3,500, 3,000, 1,600, 1,400, 1,000, 800, 500 cm^−1^. The B-granules of ZM18 and YZ4110 had less amylose content, although the difference among the total amylose contents of the three unfractionated starches was not significant. X-ray diffraction (XRD) patterns showed predominantly A-type crystallinity for all the starches. The A-granules showed sharper XRD patterns than the other starches. DSC analysis showed that the A-granules had broader ranges of gelatinization temperatures than the B-granules from the same wheat variety. The gelatinization enthalpy (ΔH) of A-granules was higher than that of B-granules. AK58 exhibited the smallest enthalpy, while ZM18 showed the largest enthalpy. In pasting tests, the A-granule starch of AK58 had higher peak, final and setback viscosity, lower breakdown and pasting temperature, and the B-granule starch and unfractionated starch of AK58 had lower peak, breakdown, final and setback viscosity and higher pasting temperature than ZM18 and YZ4110.

## 1. Introduction

Starch constitutes the major carbohydrate in the endosperm of wheat grains and serves as a multifunctional ingredient for the food or nonfood industries [1]. Starch is composed of two types of polysaccharide molecules, amylose and amylopectin. During grain development, starch is deposited in the endosperm as discrete semicrystalline aggregates known as starch granules [2]. It is widely acknowledged that in mature wheat grains starch is deposited in two distinct types of granules: A-type granules (diameter > 9.9 µm) and B-type granules (diameter < 9.9 µm) [3]. These two types of starch granules are not restricted to wheat. Dündar *et al*. demonstrated that potato starch also appears to comprise larger and smaller starch granules [4]. The large, lenticular A-type granules and the small, spherical B-type granules have different physical, chemical, and functional properties [5]. Starch granule size distribution from seven wheat cultivars under different water regimes had been investigated and it was reported that the diameter of starch granules was 0.37–52.6 μm in mature grains [6]. These differences result in the two starch granule types finding different applications in industrial food and nonfood applications. Soh *et al*. [7] concluded that an increase in the B-type granule content increased farinograph water absorption and improved pasta quality. The functional properties of starches are important for their use in food products and industrial applications, especially the pasting properties and gelatinization and retrogradation characteristics [8].

Wheat varieties are being developed for different qualities in accordance with the development of genetic recombinations [9]. In our research three cultivars, namely AK58, ZM18 and YZ4110, had proved to be improved crops. Among the three cultivars, AK58 showed in trials performed over the past few years (from 2005 to 2011) outstanding drought-enduring, freeze resistence, wide adaptability and high yield behaviors. Therefore, the objective of the present study was to characterize the A-granule and B-granule starches from three wheat cultivars by scanning electron microscopy (SEM), Fourier transform infrared spectroscopy (FTIR), amylose content analysis, X-ray diffraction (XRD), differential scanning calorimetry (DSC) and rapid viscoanalysis (RVA).

## 2. Results and Discussion

### 2.1. SEM Analysis

**Figure 1:**
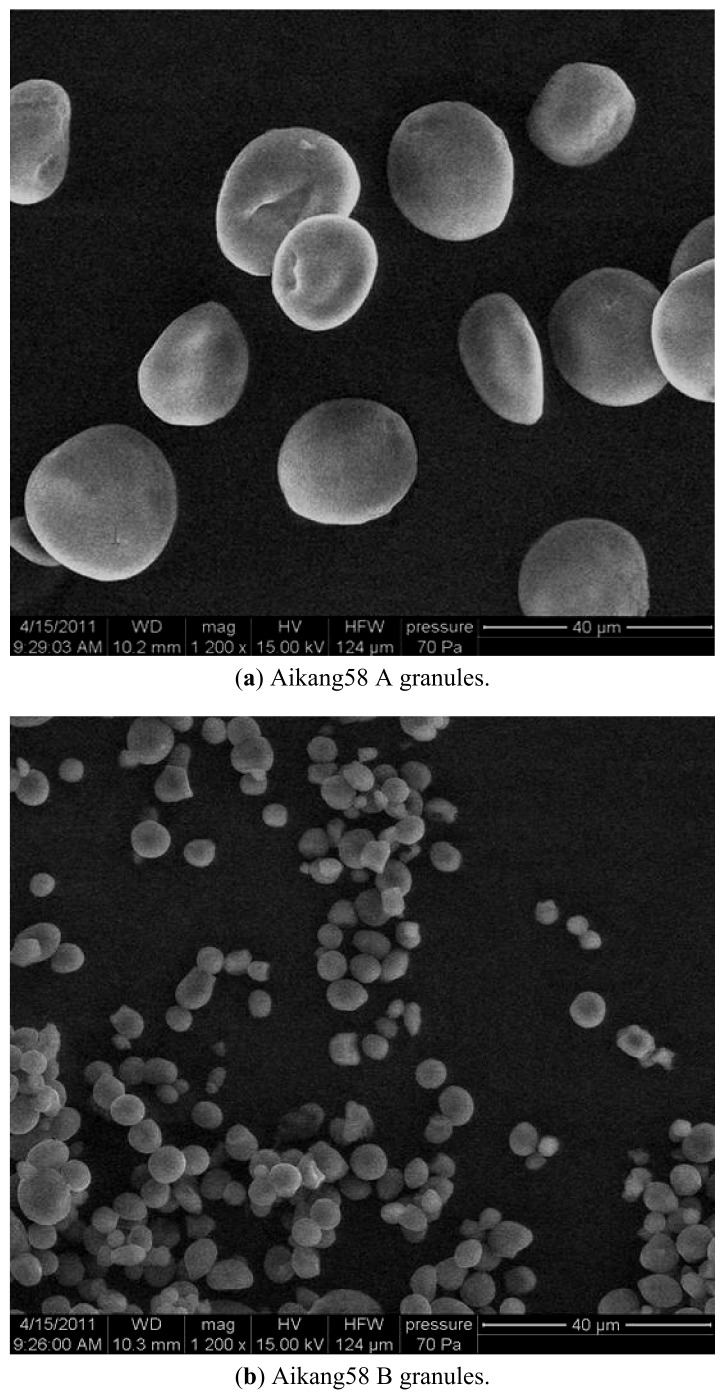

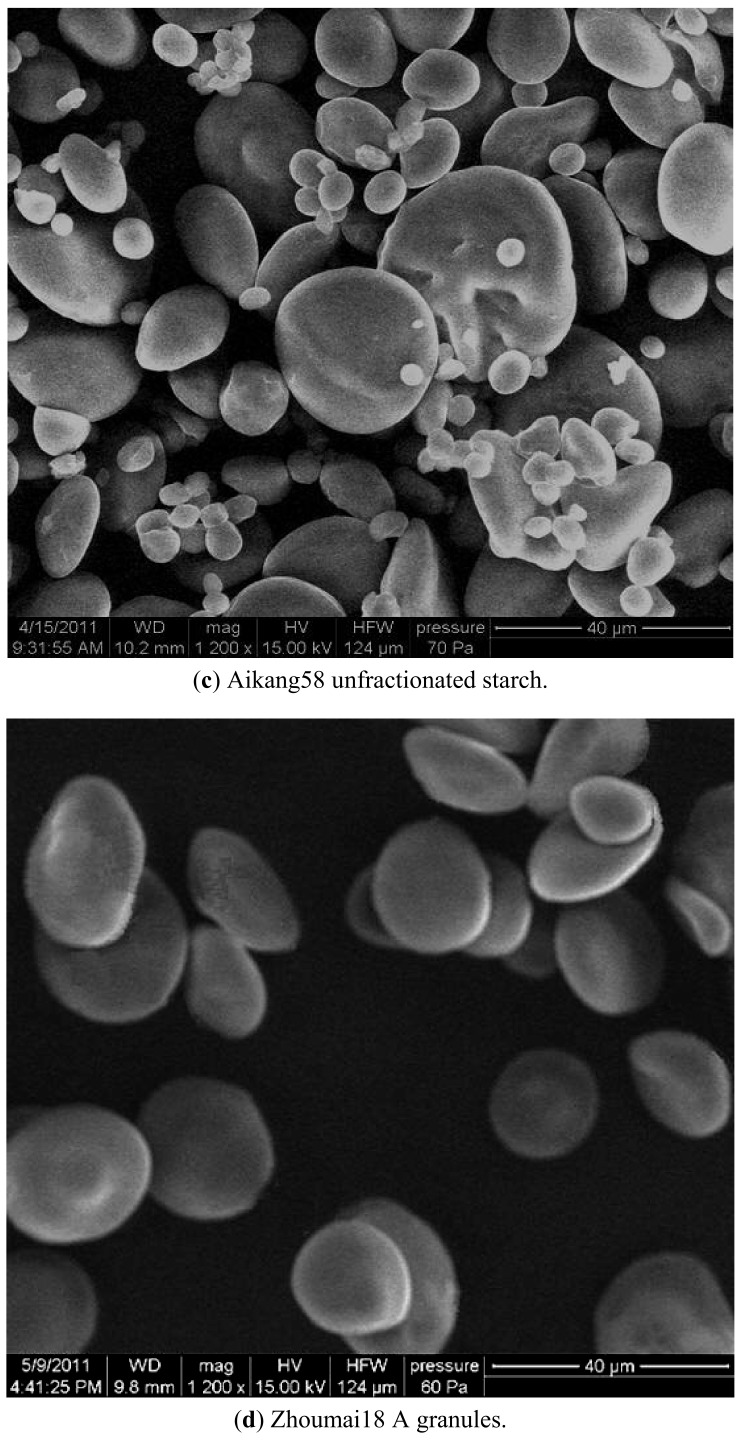

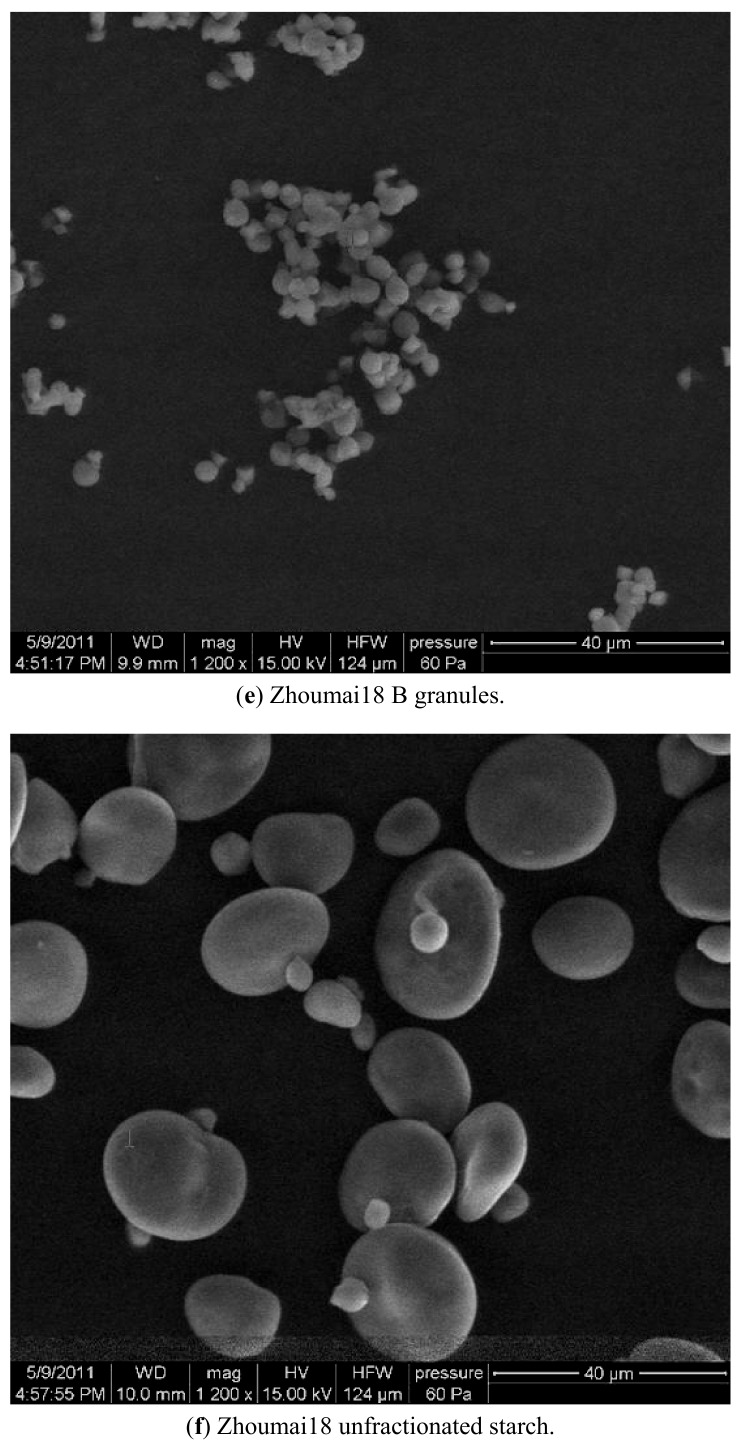

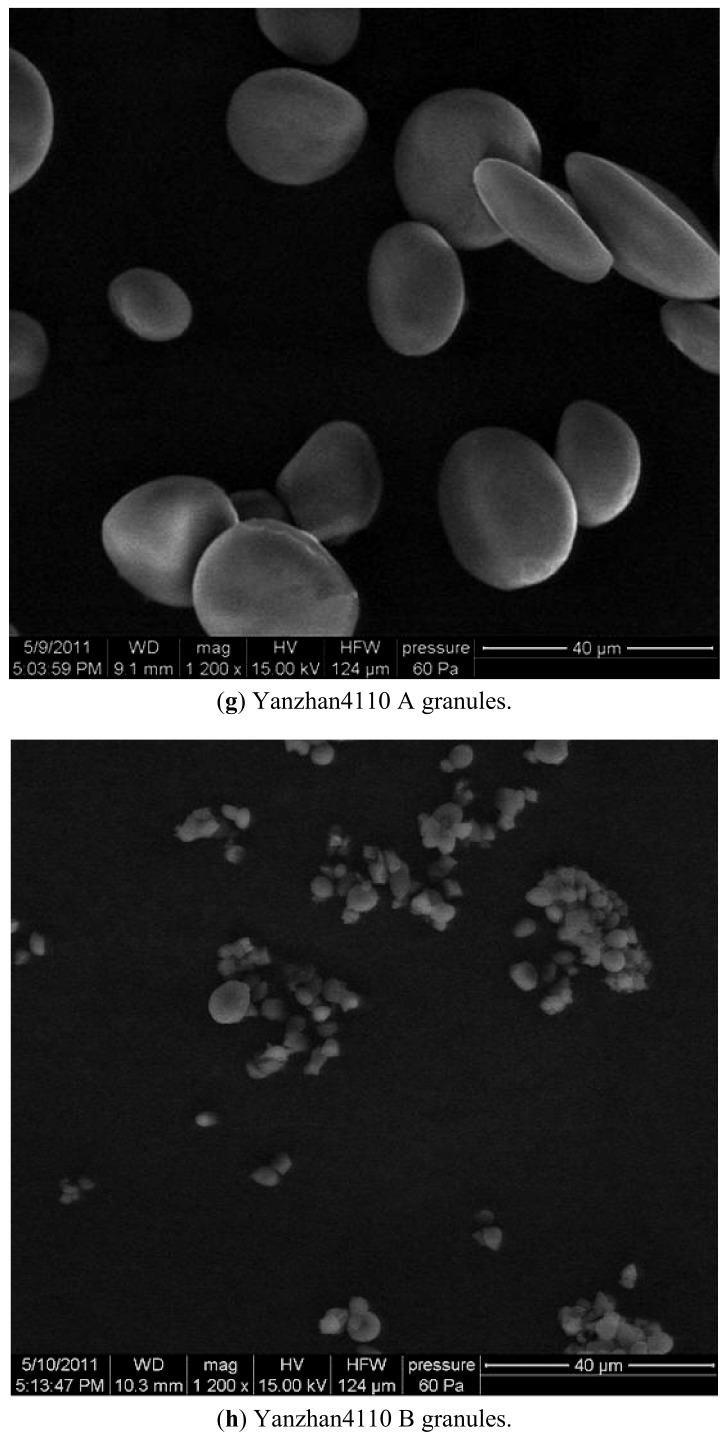

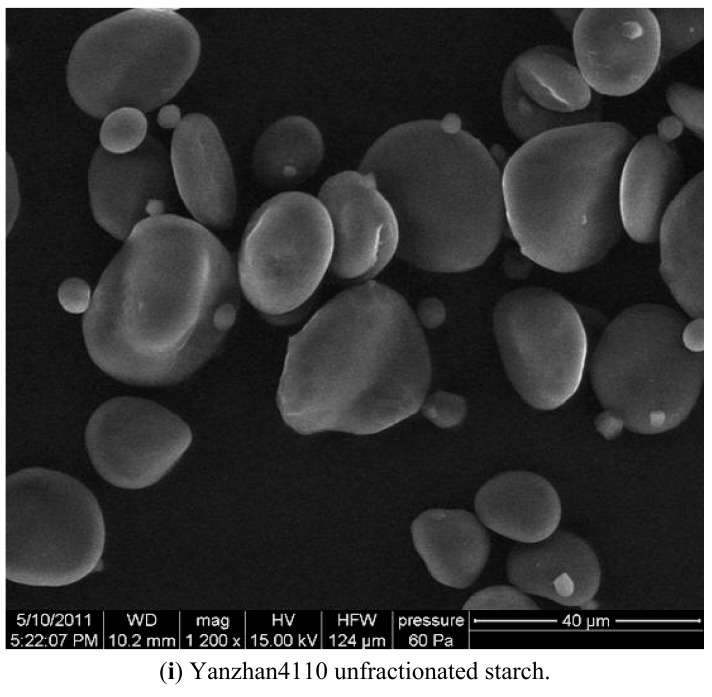
Scanning electron microscope images of unfractionated starch and isolated A- and B-granules (1200×).

The three kinds of unfractionated wheat starch granules showed bimodal size distributions (Figure 1c, f and i, respectively). The scanning electron micrographs of the A- and B-granules isolated from these three starches are shown in Figure 1. The A-starch granules displayed a disk shape with diameters of 16–28, 14–25 and 12–24 µm (Figure 1a, d, and g for AK58, ZM18 and YZ4110, respectively), while the isolated B-starch granules displayed a spherical shape with diameters of about 2.5–8.5, 1–5 and 2.5–6 µm (Figure 1b, e, and h for AK58, ZM18 and YZ4110, respectively). The SEM analysis of the starch granules was in agreement with the previous reports [10,11]. The scanning electron micrographs showed that wheat starch had a greater proportion of small granules. The size of the A-granules from the three wheat starches was nearly the same, but the size of B-granules from AK58 was larger than that of ZM18 and YZ4110.

### 2.2. FT-IR Analysis

**Figure 2:**
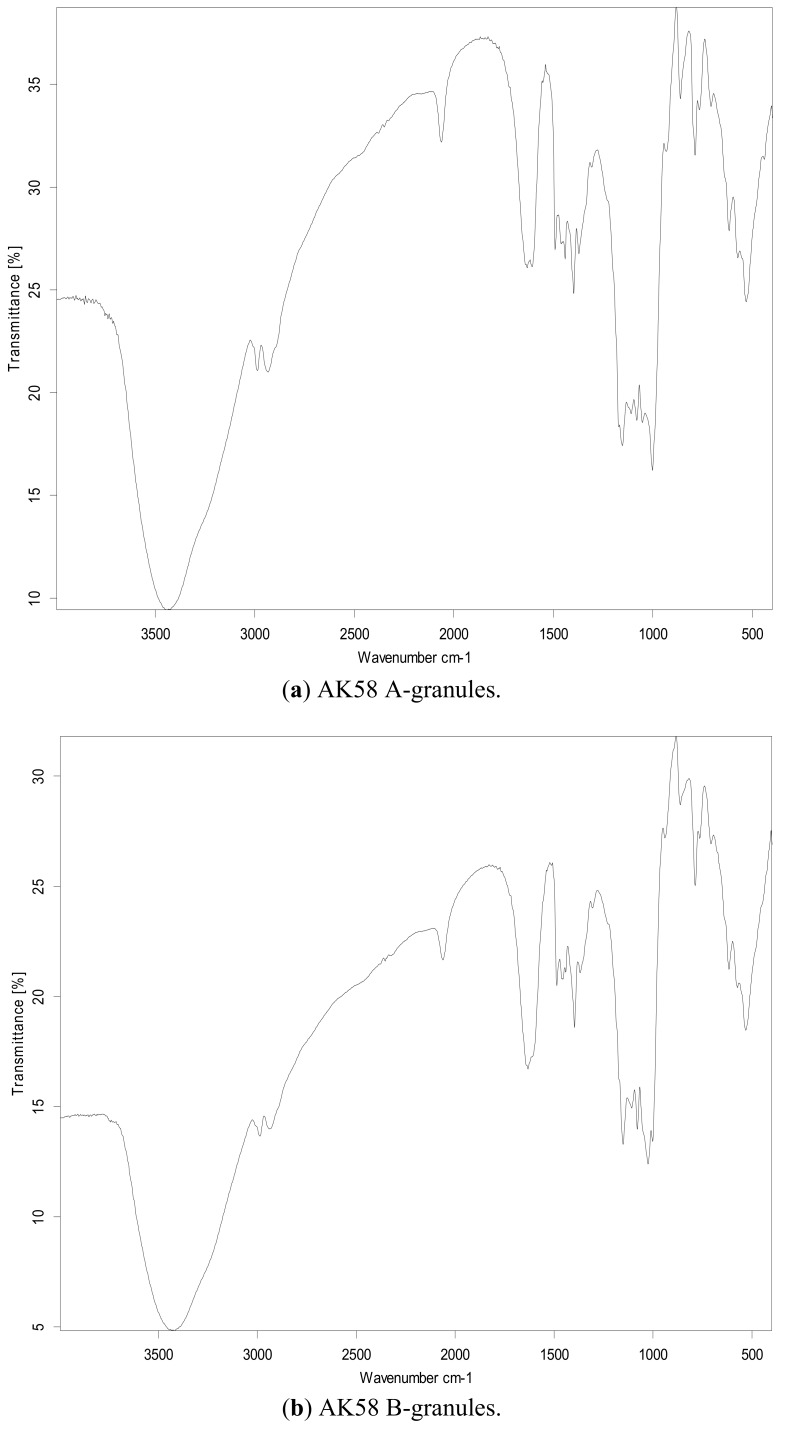

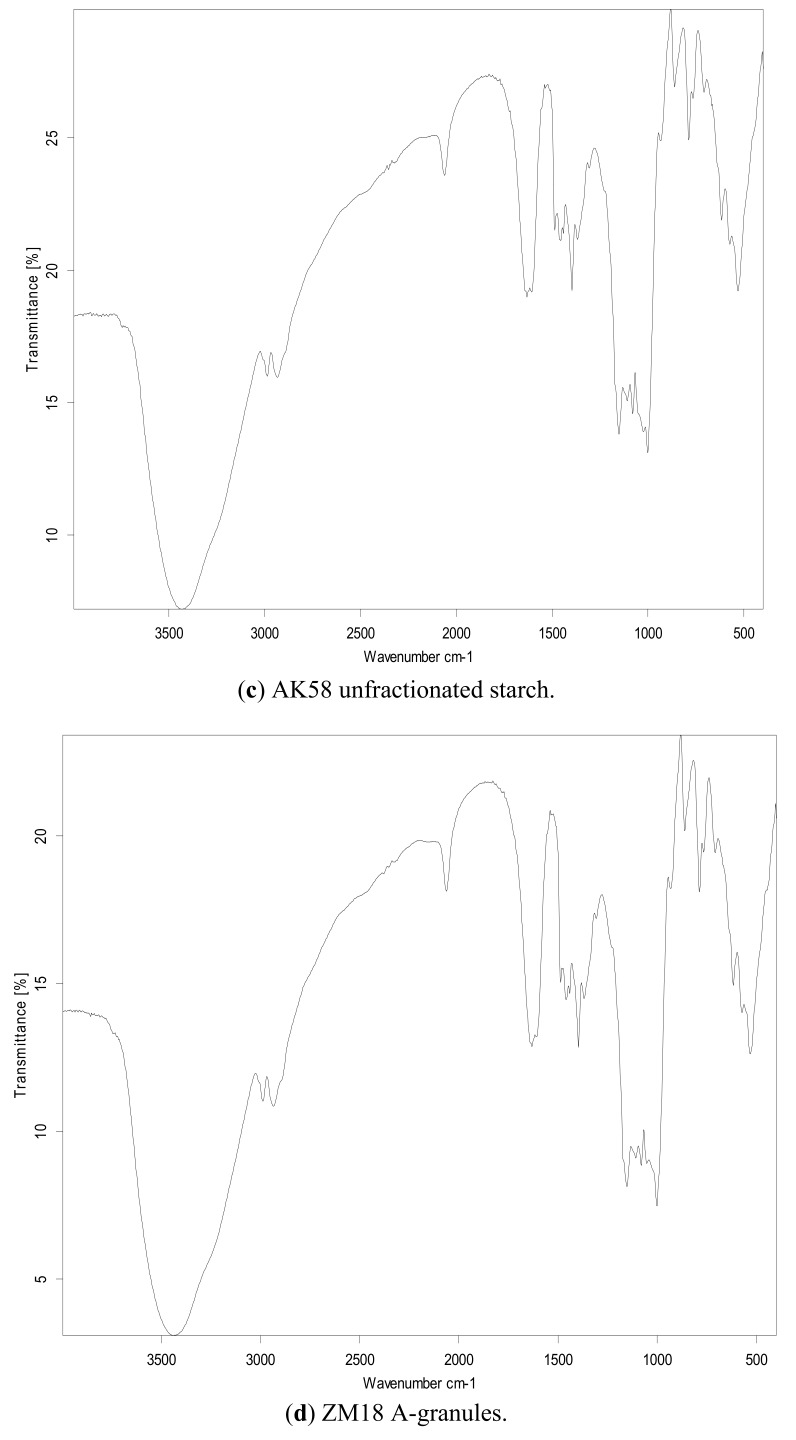

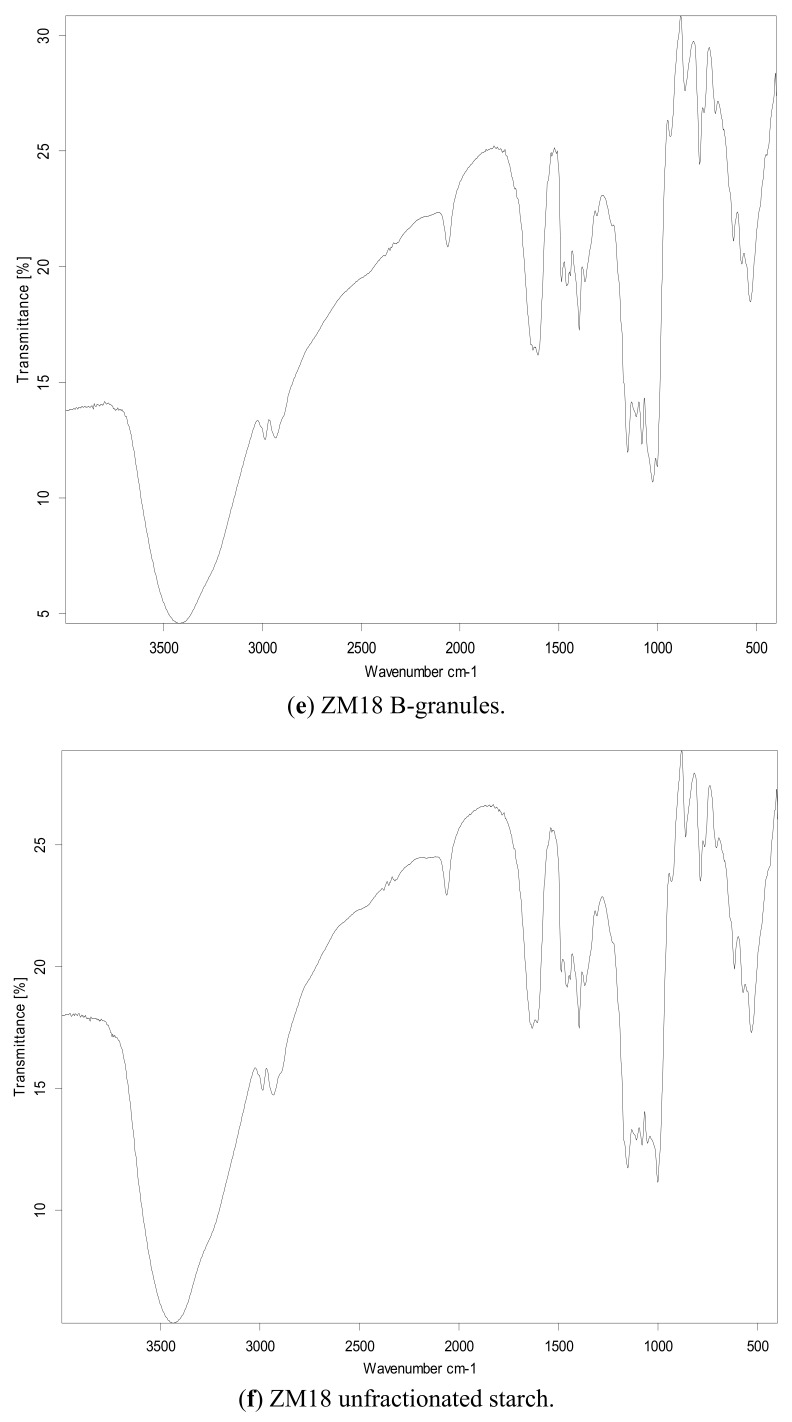

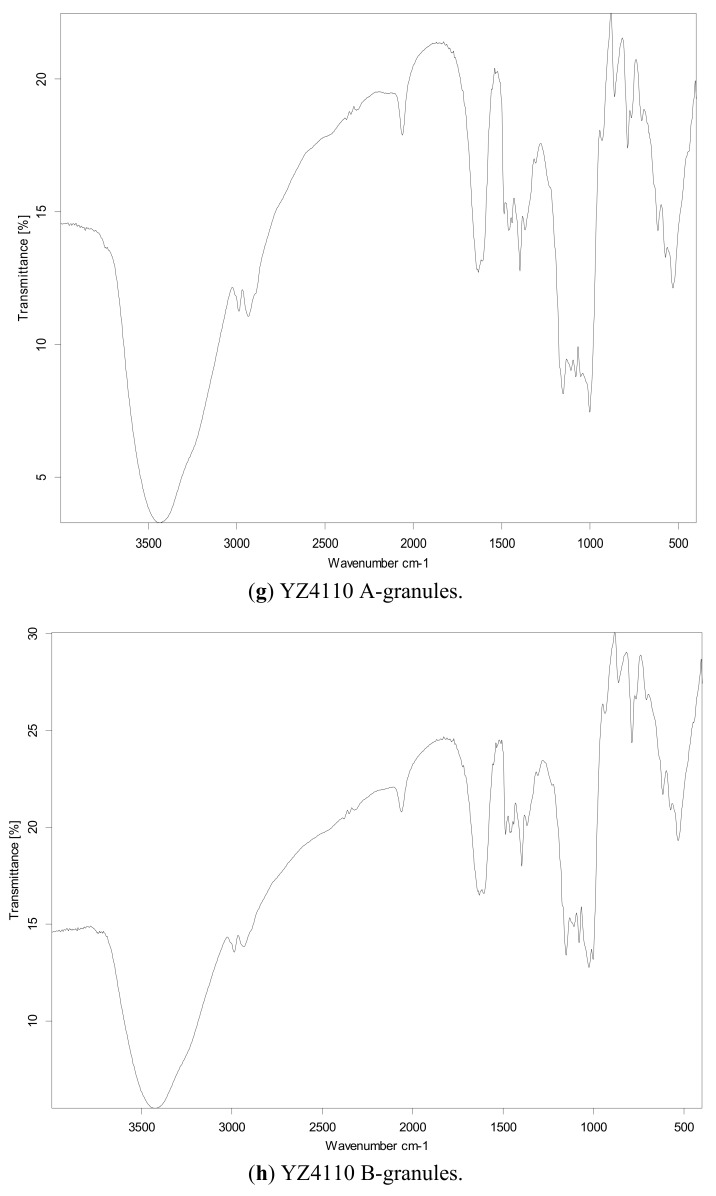

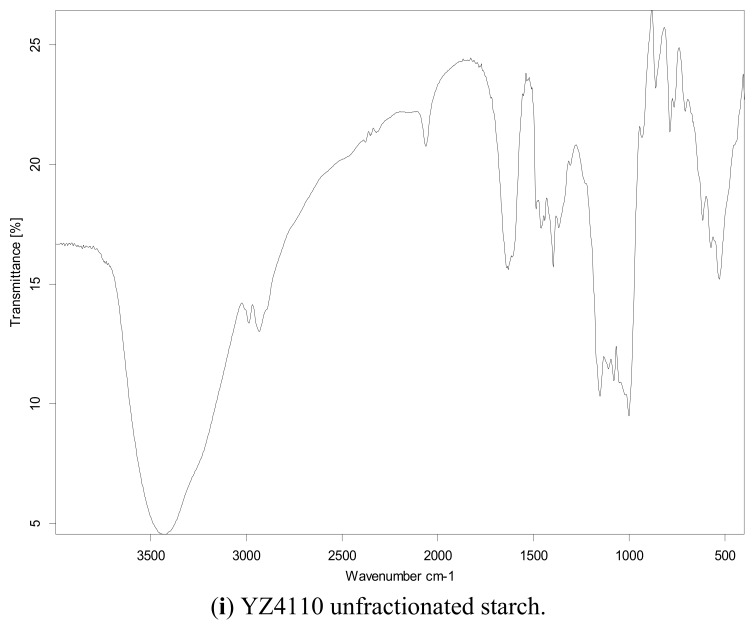
Infrared spectra of unfractionated starch and isolated A- and B-granules.

Figure 2 shows FTIR spectra of the samples in the 4,000–500 cm^−1^ region. In the previous study, on unfractionated starch, isolated A- and B-granules exhibited a similar pattern and showed 13 peaks in the 4,000–500 cm^−1^ region. The IR spectrum of starch samples was described by seven main modes, with maximum absorbance peaks near 3,500, 3,000, 1,600, 1,400, 1,000, 800 and 500 cm^−1^ [12,13]. The band absorbances in starch have been assigned and matched with the vibrational modes of the chemical bonds and the structures of starch molecules by many researchers.

The peaks at 3,405 cm^−1^ and 2,930 cm^−1^ could be attributed to O–H and C–H bond stretching, respectively, while the peaks at 1,420 cm^−1^ and 1,366 cm^−1^ were attributable to the bending modes of H–C–H, C–H and O–H. The peaks at 1,300~1,000 cm^−1^ were attributed to C–O–H stretching. The peaks at 1,155 cm^−1^, 1,097 cm^−1^ and 1,019 cm^−1^ were assigned as the C–O bond stretching. The bands at 1,047 and 1,022 cm^−1^ were associated with the ordered and amorphous structures of starch, respectively. The ratio of absorbances 1,047/1,022 cm^−1^ was used to quantify the degree of order in starch samples [14]. The bands at 930~900 cm^−1^ were attributed to D-glucopyranosyl ring vibrational modes, 844 ± 10 cm^−1^ to the C–H absorbances of the D-glucopyranosyl rings and 766 ± 10 cm^−1^ to D-glucopyranosyl ring stretching. The 1,670~1,600 cm^−1^ bands were assigned to H_2_O bending vibrations.

### 2.3. Amylose Content Analysis

**Figure 3:**
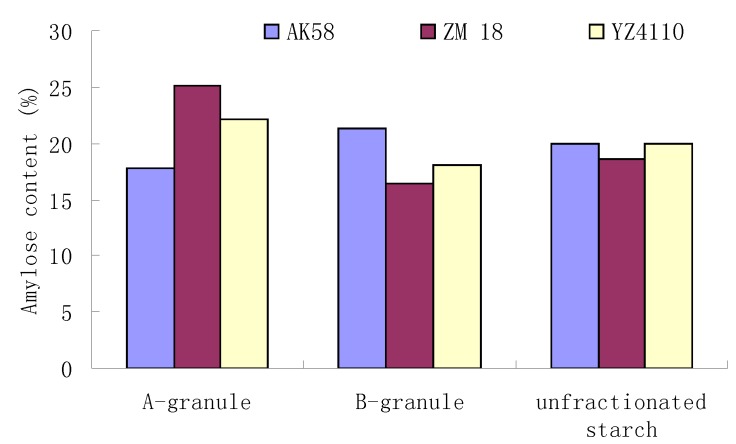
Amylose content of wheat starches and their A- granules and B-granules.

Amylose contents of the A-granules, B-granules and unfractionated starches were analyzed and the results are shown in Figure 3. The differences among the amylose contents of unfractionated starches was not significant. The amylose content of the B-granules from AK58 was higher than that of ZM18 and YZ4110, while the amylose content of the A-granules from AK58 was lower than that of ZM18 and YZ4110. For the same variety, the amylose content of B-granules from AK58 was higher than that of A-granules, while the other two wheat starches showed the opposite situation. In general, the amylose content of starch is proportional to the granule size and maturity of starch [15]. Because amylose is amorphous in the starch granule, the larger amylose content of the B-granule starch is likely to result in a lesser percentage of the crystallinity of the B-granule starch than the A-granule starch.

### 2.4. XRD Analysis

**Figure 4:**
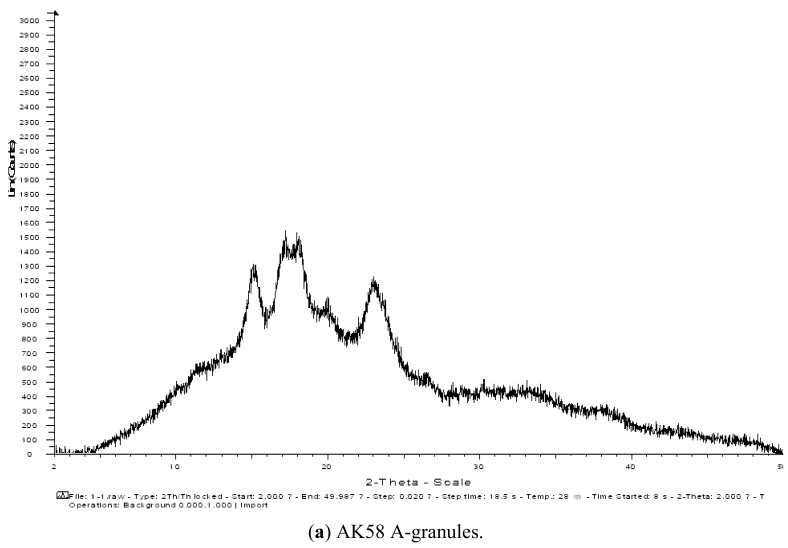

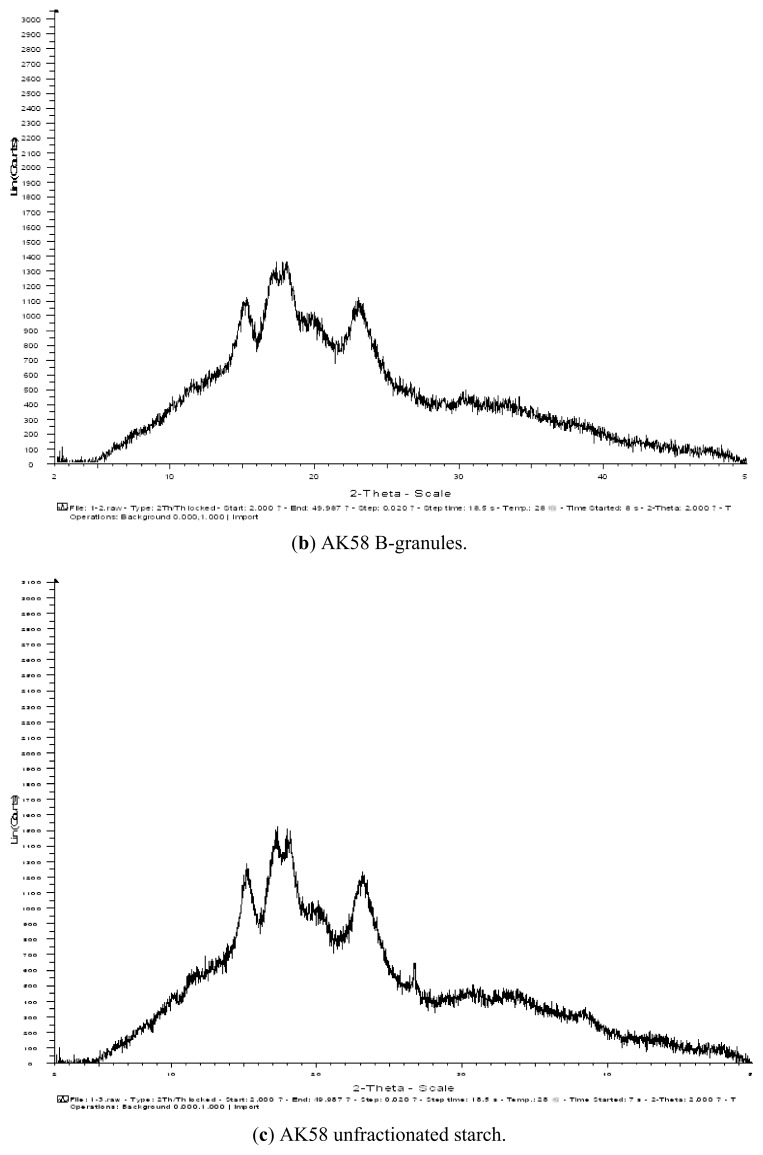

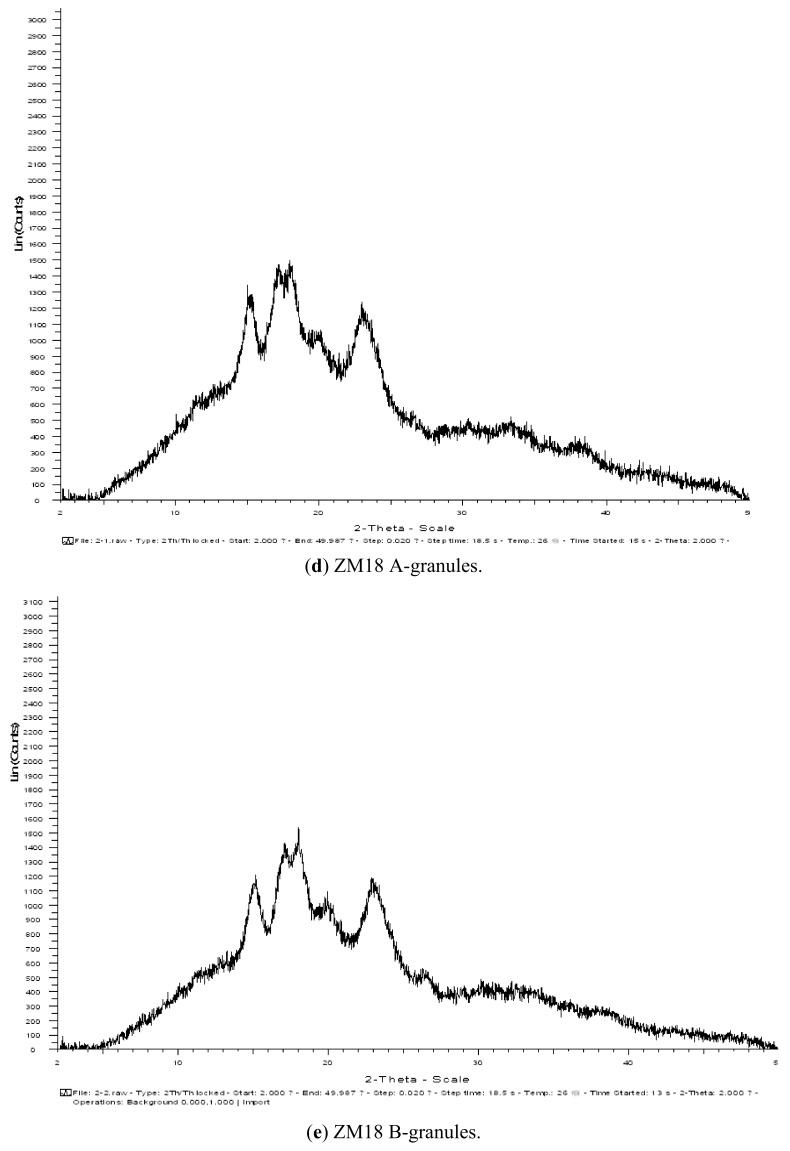

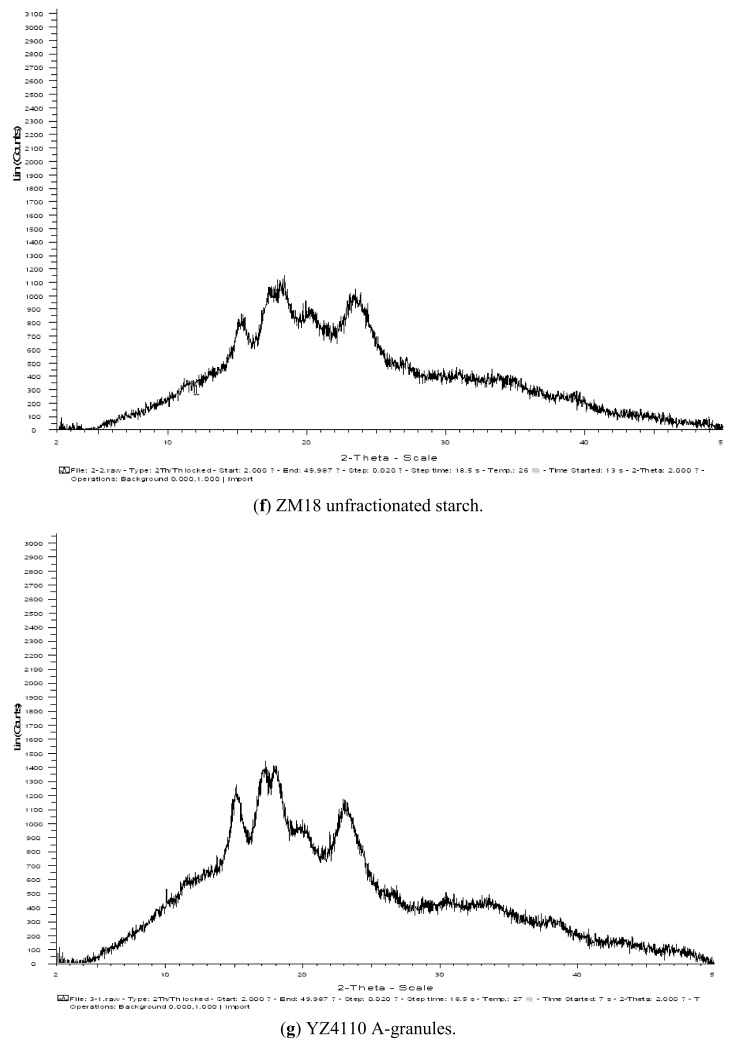

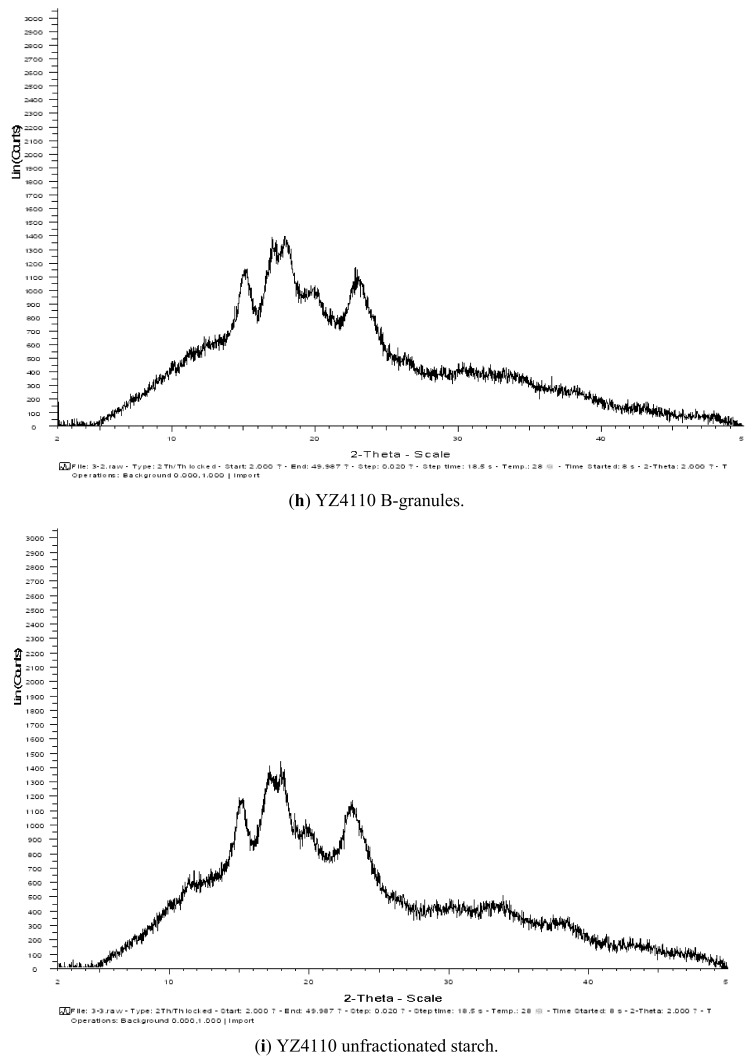
XRD profiles of unfractionated starch and isolated A- granules and B-granules.

Its X-ray diffraction pattern is the “fingerprint” of the crystal structure within starch grains. According to the characteristic X-ray diffraction lines, the crystal structure of starch can be divided into four types, including A, B, C and V type. Of these A, B, and C-type are the crystal structures of natural starch, and V type is crystalline and typical of the complexes formed by amylose and lipids. The X-ray diffraction patterns of the starches are shown in Figure 4. The unfractionated starches and the separated A- and B-granules of wheat starches all displayed typical A-type X-ray diffraction patterns at 2θ with the first peak around 15°, the second peak near 18°, and the third main reflection around 23°. In general, the XRD intensities of the unfractionated starches, and the corresponding A-granules and B-granules were almost identical to each variety. The A-granules showed sharper XRD patterns than the other starches.

### 2.5. DSC Analysis

Gelatinization properties of the starches are shown in Table 1. The A-granules had broader ranges of gelatinization temperatures (Tc-To) than the B-granules, which did not agree with the previous report that the small granule starch had a broader gelatinization temperature range than the unfractionated starch [16]. The gelatinization peak temperature (Tp) of the B-granules was higher than that of the A-granules. The gelatinization enthalpy (△H) of A-granules was higher than that of B-granules. As for A-granule starch, B- granule starch and unfractionated starch, AK58 exhibited the smallest enthalpy, while ZM18 showed the largest enthalpy.

**Table 1:** Thermal properties of unfractionated starch, A and B granules of wheat varieties.

Variety	Granule	To (°C)	Tp (°C)	Tc (°C)	Tc-To	△H
AK58	A	60.48	64.86	84.25	23.77	2.663
	B	64.29	71.58	85.39	21.10	1.807
	Unfractionated starch	61.13	66.05	86.34	25.21	2.289
ZM18	A	60.72	65.66	85.58	24.86	3.342
	B	66.36	73.63	88.95	22.35	2.888
	Unfractionated starch	61.48	66.91	84.25	23.02	2.933
YZ4110	A	59.65	64.01	80.65	20.85	2.869
	B	63.05	69.60	82.35	19.30	2.196
	Unfractionated starch	60.58	65.09	80.08	19.50	2.819

### 2.6. Pasting Properties

Pasting parameters of A-granule starch, B-granule starch and unfractionated starches of wheat are shown in Table 2. For AK58, the B-granule starch had the higher pasting temperature (84.75 °C) and lower breakdown and setback viscosities than the A-granule starch (77.1 °C), while the A-granule starch had the greater peak, final, breakdown, and setback viscosities than the B-granule starch counterparts.

**Table 2:** Pasting properties of the unfractionated starch, A and B granules of wheat varieties.

Variety	Granule	Peak (cP)	Trough (cP)	Breakdown (cP)	Final viscosity (cP)	Setback (cP)	Pasting Temperature (°C)
AK58	A	3750	3230	529	4576	1355	77.1
	B	3345	3033	312	3765	732	84.75
	Unfract. starch	3237	2785	452	3927	1143	84.45
ZM18	A	3200	2507	710	3698	1207	85.75
	B	3804	3226	578	4025	799	76.51
	Unfract. starch	3788	2723	1065	4475	1752	78.67
YZ4110	A	3312	2677	635	3816	1139	84.95
	B	3376	2888	488	4340	1452	79.30
	Unfract. starch	3347	2751	597	4191	1441	81.38

For ZM18, the B-granule starch had lower pasting temperature, (76.51 °C), breakdown and setback viscosities and higher peak and final viscosities than did the A-granule starch, while the A-granule starch had greater pasting temperature (85.75 °C) than the B-granule starch.

For YZ4110, the difference of peak viscosities among A-granule, B-granule and unfractionated starch was no obvious. The B-granule starch had the higher final viscosity and setback and than the A-granule starch and unfractionated starch, while the A-granule starch had the greater breakdown and pasting temperature than the B-granule starch and unfractionated starch.

The differences in the pasting properties among three different wheat starches was significant. As shown in Table 2, the A-granule starch of AK58 had the higher peak, final and setback viscosity and lower breakdown and pasting temperature than that of ZM18 and YZ4110. The B-granule starch and unfractionated starch of AK58 had the lower peak, breakdown, final and setback viscosities and higher pasting temperature than that of ZM18 and YZ4110.

## 3. Experimental

### 3.1. Materials and Chemicals

Three wheat varieties (AK58, YZ4110 and ZM18) were used in this study. They were provided as grains by the Wheat Research Center of Henan Institute of Science and Technology. The wheat grain was milled to straight-grade flour in a Senior mill (zs70-Ⅱ, Zhuozhou Cereal and Oil Machinery Factory, China) according to the approved method 26–31 (AACC, 2000). Other reagents and chemicals used were at minimum of analytical grade.

### 3.2. Isolation of Starch

The wheat starches were isolated following the method reported by Kasemsuwan *et al*. [17]. The isolated starches were washed with water and ethanol, and recovered by filtration using filter paper before drying in a convection oven at 32 °C for 48 h.

### 3.3. Separation of the A-and B-Granule Starch

The A-and B-granules were separated by sedimentation using graduated cylinders (2 L) as described by Takeda *et al.* [18]. The fraction of 2 h precipitate was collected as large granules (A-granules) and the fraction remaining in the supernatant after 20-h sedimentation was collected as small granules (B-granules). The fractionation processes were repeated five times for the A-granules and three times for the B-granules. The separated A-and B-granule suspensions were centrifuged at 7,000 rpm for 20 min and washed with three volumes of ethanol one time. These starches were recovered by filtration (Whatman No.4 filter paper) and then dried in a convection oven at 32 °C for 48 h. Microscopic images showed no contamination of granules of other sizes in each fraction.

### 3.4. Amylose Content Determination

Amylose content of the sample was determined by using the method of Yoo and Jane [8]. The starch sample (20 mg) and 10 mL of 0.5 N KOH were mixed. The dispersed sample was diluted to 100 mL with distilled water. An aliquot of test sample solution (10 mL), 5 mL of 0.1 N HCl and 0.5 mL of iodine reagent were mixed and diluted to 50 mL. Then the absorbance of the mixture was measured at 625 nm. The amylose content was determined from a standard curve with amylose and amylopectin blends as standard.

### 3.5. SEM

Scanning electron microscopy (SEM) micrographs was recorded with a Quanta 200 environmental scanning electron microscope (FEI Company, Hillsboro, OR, USA). The samples were evenly distributed on SEM specimen stubs with double adhesive tape. The micrographs were obtained with an accelerating potential of 15 kV under low vacuum. The micrographs obtained were used to detect any damage to the starch granules [12].

### 3.6. FT-IR

In order to further determine the structure of the wheat starches, the FT-IR spectra were obtained using FT-IR (Nicolet 470; Perkin Elmer Inc., Waltham, MA, USA). The spectra were recorded in transmission mode from 4,000 to 500 cm^−1^ (mid-infrared region) at a resolution of 0.44 cm^−1^. The sample was diluted with KBr (1:100, w/w) before acquisition and the background value from pure KBr was acquired before the sample was scanned.

### 3.7. XRD

Monochromatic Cu Ka radiation (wavelength = 1.54056 Å) was produced by a D/MAX 2500V/PC X-ray diffractometer (Rigaku Americas Corporation, Salem, NH, USA). The samples were incubated in a chamber at 100% RH for 24 h and then packed tightly in a rectangular aluminum cell. The samples were exposed to the X-ray beam from an X-ray generator running at 36 kV and 20 mA. The scanning regions of the diffraction angle, 2θ, were 0–30°, which covered most of the significant diffraction peaks of the starch crystallites. Other operation conditions included: step interval 0.02, scan rate 4°/min, Sollet and divergence slit 1°, receiving slit 1°, and scattering slit 0.16°. Duplicate measurements were made at ambient temperature. Radiation was detected with a proportional detector.

### 3.8. DSC

Thermal properties of the starches were analyzed using a differential scanning calorimeter (DSC, TA instruments Waters LLC, New Castle, DE, USA) equipped with a thermal analysis data station. Aluminum pans (Perkin-Elmer) were used for the analysis. Starch samples (2 mg, dried starch basis, dsb) were precisely weighed in the sample pans, mixed with distilled water (4 mg), and sealed. The heating rate was at 10 °C per min over the temperature range of 30–120 °C. Indium and zinc were used as the reference standards. Enthalpy change (△H), gelatinization onset temperature (To), peak temperature (Tp), and conclusion temperature (Tc) were measured. The data were averages of a minimum of three replicates of each starch sample.

### 3.9. RVA

Pasting properties of the starch were determined by using a Rapid Visco-Analyzer (RVA) (RVA-Series 4, Newport Scientific Pty. Ltd., Warriewood, Australia). Each starch suspension (8%, w/w; 28 g total weight) was equilibrated at 50 °C for 1 min and then heated at a rate of 6 °C/min to 95 °C and then maintained at that temperature for 5 min. The sample was then cooled to 50 °C at a rate of 6 °C/min. A rotating speed of the paddle (160 rpm) was used except the paddle speed was 960 rpm at the first 10 s.

### 3.10. Statistical Analysis

The data obtained in this study were expressed as the mean of three replicate determinations and standard deviation (SD). Statistical comparisons were carried out using student t test. P values of <0.05 were considered to be significant.

## 4. Conclusions

SEM results showed that the size of A-granules from ZM18 and YZ4110 were about the same, but the sizes of A-granules (16–28 µm) and B-granules (2.5–8.5 µm) from AK58 were larger than those of ZM18 and YZ4110. FTIR spectra showed that unfractionated starch and isolated A- and B- granules exhibited a similar pattern. The total amylose contents of the three unfractionated starches were about the same, but the A-granule starch of AK58 contained less amylose than that of ZM18 and YZ4110. XRD patterns showed predominantly A-type crystallinity for all of the starches. The A-granules possessed sharper XRD patterns than the B-granules. The A-granules had the broader ranges of gelatinization temperatures than the B-granules and the gelatinization enthalpy (△H) of A-granule was higher than that of B-granule starches. AK58 exhibited the smallest enthalpy while ZM18 showed the largest enthalpy. The A-granules starch of AK58 had higher peak, final and setback viscosity and lower breakdown and pasting temperature, and the B-granule and unfractionated starch of AK58 had lower peak, breakdown, final and setback viscosity and higher pasting temperature than that of ZM18 and YZ4110. These results showed that the structures and properties of the A- and B-granule starches were distinct, which suggested that biosynthesis of the A- and the B-granules differed.

## Supplementary Materials

Supplementary File 1

## References

[1] Li W.Y., Yan S.H., Yin Y.P., Li Y., Liang T.B., Gu F., Dai Z.M., Wang Z.L. (2008). Comparison of starch granule size distribution between hard and soft wheat cultivars in eastern China. Agric. Sci. China.

[2] Paul C. (1997). The structure of starch. Nature.

[3] Soulaka A.B., Morrison W.R. (1985). The amylose and lipid content, dimensions, and gelatinization charactertistics of some wheat starches and their A- and B-granule fractions. J. Sci. Food Agric..

[4] Dündar E., Turan Y., Blaurock A.E. (2009). Largescale structure of wheat, rice and potato starch revealed by ultra small angle X-ray diffraction. Int. J. Biol. Macromol..

[5] Ellis R.P., Cochrane M.P., Dale M.F.B., Duffus C.M., Lynn A., Morrison I.M., Prentice R.D.M, Swanston J.S., Tiller S.A. (1998). Starch production and industrial use. J. Sci. Food Agric..

[6] Dai Z., Yin Y., Wang Z. (2009). Starch granule size distribution from seven wheat cultivars under different water regimes. Cereal Chem..

[7] Soh H.N., Sissons M.J., Turner M.A. (2006). Effect of starch granule size distribution and elevated amylase content on durum dough rheology and spaghetti cooking quality. Cereal Chem..

[8] Šubarić D., Babić J., LaLić A., Ačkar D., Kopjar M. (2011). Isolation and characterization of starch from different barley and oat varieties. Czech J. Food Sci..

[9] Singh N., Singh S., Isono N., Noda T., Singh A.M. (2009). Diversity in amylopectin structure, thermal and pasting properties of starches from wheat varieties/lines. Int. J. Biol. Macromol..

[10] Jane J., Kasemsuwan T., Leas S., Zobel H., Robyt J.F. (1994). Anthology of starch granule morphology by scanning electron microscopy. Starch/Staerke.

[11] Yoo S.H., Jane J. (2002). Structural and physical characteristics of waxy and other wheat starches. Carbohyd. Polym..

[12] Koksel H., Masatcioglu T., Kahraman K., Ozturk S., Basman A. (2008). Improving effect of lyophilization on functional properties of resistant starch preparations formed by acid hydrolysis and heat treatment. J. Cereal Sci..

[13] Sitohy M.Z., Labib S.M., El-Saadany S.S., Ramadan M.F.  (2000). . Optimizing the conditions for starch dry phosphorylation with sodium mono- and dihydrogen orthophosphate under heat and vacuum. Starch/Staerke.

[14] Goni I., García-Diz L., Mañas E., Saura-Calixto F. (1996). Analysis of resistant starch: a method for foods and food products. Food Chem..

[15] Pan D.D., Jane J. (2000). Internal structure of normal maize starch granules revealed by chemical surface gelatinization. Biomacromolecules.

[16] Eliasson A.C., Karlson R. (1983). Gelatinization properties of different size classes of wheat starch granules measured with differential scanning calorimetry. Starch/Staerke.

[17] Kasemsuwan T., Jane J., Schnable P., Stinard P., Robertson D. (1995). Characterization of the dominant mutant amylose-extender (Ael-5180) maize starch. Cereal Chem..

[18] Takeda Y., Takeda C., Mizukami H., Hanashiro I. (1999). Structures of large, medium and small starch granules of barley grain. Carbohyd. Polym..

