# Supplementary material for: Comparison of A and B Starch Granules from Three Wheat Varieties
Source: Molecules. 2011 Dec 19;16(12):10570–91. doi: 10.3390/molecules161210570 (PMC6264545; doi:10.3390/molecules161210570)
Supplement: Supplementary File 1 [file molecules-16-10570-s001.pdf]

Molecules **2012**, *17*

OPEN ACCESS

molecules

ISSN 1420-3049

www.mdpi.com/journal/molecules

Correction

Ru *et al.* Comparison of A and B Starch Granules from Three Wheat Varieties. *Molecules*, 2011, *16*, 10570-10591

Jie Zeng [1], Guanglei Li [1], Haiyan Gao [1] and Zhengang Ru [2,*]

[1] School of Food Science, Henan Institute of Science and Technology, Xinxiang 453003, China; E-Mails: zengjie623@163.com (J.Z.); lgl70_hist@163.com (G.L.); gaohaiyan127@163.com (H.G.)

[2] Wheat Research center, Henan Institute of Science and Technology, Xinxiang 453003, China

* Author to whom correspondence should be addressed; E-Mail: ruzhengang2011@163.com; Tel.: +86-373-369-3005; Fax: +86-373-304-0709.

Received: 27 April 2012; in revised form: 27 April 2012 / Accepted: 2 May 2012 / Published: 2 May 2012

The authors wish to make the following correction to this paper [1]:

The correct Program Number is 2011BAD07B02, therefore the Acknowledgments should read as follows: This study was funded by the Henan Province Special Funds for Major Scientific and Technological Innovation (111100110100), National Key Basic Research Development Program (973 Program, 2012CB114300) and National Science & Technology Pillar Program (2011BAD07B02).

Reference

1. Zeng, J.; Li, G.; Gao, H.; Ru, Z. Comparison of A and B Starch Granules from Three Wheat Varieties. *Molecules* **2011**, *16*, 10570–10591.

© 2012 by the authors; licensee MDPI, Basel, Switzerland. This article is an open access article distributed under the terms and conditions of the Creative Commons Attribution license (http://creativecommons.org/licenses/by/3.0/).
